# Ist die elektive Bestrahlung der Lymphknotenlevel IV und Vb bei Nasopharynxkarzinomen erforderlich?

**DOI:** 10.1007/s00066-022-01986-3

**Published:** 2022-08-11

**Authors:** Sabine Semrau, Marlen Haderlein

**Affiliations:** Strahlenklinik, Friedrich-Alexander-Universität Erlangen-Nürnberg, Universitätsstr. 27, 91054 Erlangen, Deutschland

## Hintergrund und Ziele

Das Ausmaß der Bestrahlung von makroskopisch tumorfrei imponierenden Lymphknotenstationen bei Nasopharynxkarzinomen ist Gegenstand der Diskussion seit mehr als einem Jahrzehnt. Selbst Leitlinien sind zurückhaltend in der Formulierung, ob kaudale Lymphknotenlevel ausgespart oder bei ausschließlichem Befall kranial gelegener Lymphknotenlevel einbezogen werden sollten [1]. Untersuchungen der Studiengruppe aus Guangzhou im Jahr 2009 mittels MRT hatten ergeben, dass es zu einem schrittweisen Befall zunächst der retropharyngealen Lymphknoten gefolgt von den Regionen II, III, Va und erst später der Level IV und Vb kommt. Die Wahrscheinlichkeit des „Überspringens“ einer Lymphknotenstation wurde seinerzeit mit 0,5 % ermittelt [2]. Die eindrückliche Akut- und Langzeittoxizität der totalen bilateralen pharyngealen bzw. zervikalen Bestrahlung nahezu aller Lymphknotenlevel induziert das Bedürfnis, das Bestrahlungsvolumen differenzierter festzulegen. Aus der Studiengruppe um Ling-Long Tang wurde deshalb eine randomisierte Studie zur Frage durchgeführt, ob bei einem Patientenkollektiv mit limitierter Lymphknotenmetastasierung eine auf die oberen Lymphknotenstationen beschränkte Bestrahlung zu einer vergleichbaren regionären Tumorkontrolle führt wie die Bestrahlung der gesamten Lymphabflussgebiete beider Halsseiten.

## Patientengut und Methoden

Eingeschlossen wurden chinesische Patienten mit einem nicht verhornenden Plattenepithelkarzinom, gleichbedeutend mit dem in Asien endemischen Schmincke-Tumor, mit einem limitierten Lymphknotenbefall bis zum Stadium cT4 cN1 nach AJCC-TNM-Klassifikation Version 7.0. Dies umfasst einen unilateralen Befall der Halslymphknoten bis 6 cm oberhalb der Supraklavikulargrube und maximal einen bilateralen retropharyngealen Befall bis 6 cm Durchmesser. (Cave: In der TNM-Version 8 ändert sich die anatomische Grenze bei cN1, nämlich nicht mehr oberhalb der Supraklavikularregion, sondern oberhalb der kaudalen Begrenzung des Cartilago cricoidea. Jeglicher Lymphknotenbefall unterhalb des Ringknorpels ist cN3.) Patienten mit einem bilateralen Befall der oberen Halslymphknoten (N2) oder Patienten mit verhornenden Plattenepithelkarzinomen oder nichtverhornenden Plattenepithelkarzinomen vom WHO-Typ 2, wie diese häufig in Europa beobachtet werden, wurden nicht in dem Protokoll behandelt oder Letztere nur in einer marginalen Anzahl (1 % im volumenreduzierten Arm, 2 % im Arm mit bilateraler LAG-Bestrahlung).

Bestrahlt wurden der gesamte Hals (Level II, III, IV, Va,b) und Pharynx je nach Befall und Befallsrisiko mit 66–70 Gy, 60–62 Gy und 54–56 Gy in 30–33 Fraktionen im Standardtherapiearm, mutmaßlich in SIB-Technik. Im Experimentalarm wurden die Level IV und Vb je nach makroskopischem Befall der Lymphknoten ausgeklammert. Auf deren Bestrahlung beidseits wurde verzichtet, wenn kein oder allein ein retropharyngealer Lymphknotenbefall vorlag. Bei einseitigem Befall nichtretropharyngealer Lymphknoten wurden nur die Level Vb und IV der Gegenseite ausgeklammert. Patienten ab dem Stadium II erhielten eine simultane Chemotherapie; jeder 4. Patient eine Induktionschemotherapie. Man nahm an, dass die im Volumen reduzierte Bestrahlung nach 3 Jahren nur maximal 8 % mehr regionale Rezidive hervorbringen würde als die Bestrahlung des gesamten Halses, um als gleichwertig zu gelten.

## Ergebnisse

Es wurden 446 Patienten randomisiert. In jeder Gruppe hatten etwas mehr als die Hälfte einen unilateralen zervikalen Lymphknotenbefall. Alle Patienten hielten die Strahlen- und Chemotherapie durch. Die Daten sind als verlässlich einzustufen, denn mindestens 95 % der Patienten wurden wenigstens drei Jahre nachbeobachtet. Die Rate an Lymphknotenrezidiven war in beiden Gruppen mit 3 % gleich. In der Gruppe mit limitierter Lymphknotenbestrahlung hatte kein Patient ein Rezidiv im ausgesparten Areal. Zwölf von 13 Patienten hatten das Rezidiv ausschließlich retropharyngeal. 6 % der Patienten entwickelten innerhalb von 3 Jahren Fernmetastasen. Sofern es Todesfälle gab, resultierten diese aus einer unbeherrschten Tumorsituation. Es wurde nur ein interkurrenter Todesfall berichtet. Nach 3 Jahren überlebten in beiden Behandlungsarmen gleich viele Patienten (99 % resp. 96 %). Die Rate der akuten Nebenwirkungen war in beiden Armen identisch. Allerdings zeigten sich signifikant seltener Langzeitnebenwirkungen bei den Patienten mit reduziertem Bestrahlungsvolumen: Weniger Patienten entwickelten eine Hypothyreose (30 % vs. 39 %), eine Dermatitis (14 % vs. 25 %), eine Dysphagie (17 % vs. 32 %) und eine Fibrose der Halsweichteile (23 % vs. 40 %). Der Unterschied fiel allerdings geringer aus, wenn nur an einer Halsseite die Level IV und Va ausgespart werden konnten. Schwerwiegende (≥ III°) Spätnebenwirkungen wurden in beiden Behandlungsgruppen nur vereinzelt beobachtet. Die Rate der Patienten mit Xerostomie blieb erwartungsgemäß unbeeinflusst. Diese eher gering anmutenden Verbesserungen hatten jedoch deutliche Auswirkungen auf die Lebensqualität der Patienten. Die Schluckfunktion war deutlich besser mit reduziertem Bestrahlungsvolumen und daraus resultierend auch der selbst eingeschätzte Gesundheitszustand, die Ermüdungstendenz und der emotionale Zustand der Patienten.

## Schlussfolgerung der Autoren

Trotz des Verzichts auf eine Bestrahlung der Level IV und Vb wurde keine Verschlechterung der Tumorkontrolle beobachtet, jedoch war die Langzeittoxizität deutlich geringer. Dies sollte Anlass sein, das bisher routinemäßig regelhaft praktizierte Vorgehen bei der Definition des Bestrahlungsvolumens zu ändern.

## Kommentar

„Essen ist der Himmel der einfachen Leute.“ Dieses Jahrtausende alte Sprichwort charakterisiert die innige Bedeutung des beschwerdefreien Essens als Lebensqualität der Asiaten. Während wir Mitteleuropäer uns einer vernünftigen Ernährung rühmen, ist den Asiaten der Genuss kräftiger Speisen heilig. Auch uns geht es tief im Inneren beim Essen nicht nur um eine bloße Nahrungsaufnahme, und ein gutes Essen in illustrer Gesellschaft ist manchem eine „Sünde“ wert. Den Gedanken an das Vergnügen des gemeinsamen Speisens zu verinnerlichen, hilft uns zu verstehen, wie vielschichtig gerade auch in Bezug auf das soziale Leben der Verlust ist, wenn man unter einer Tumortherapie weder gut riechen noch schmecken noch schlucken kann. Deshalb sind Lebensqualitätsanalysen in Studien so wichtig. Der in der Studie von Tang et al. [3] erreichte Unterschied in der Lebensqualität ist vergleichbar mit den Unterschieden in der Lebensqualität von Patienten, die beispielsweise wegen eines kleinen Tonsillenkarzinoms lediglich allein operiert wurden, verglichen mit Patienten, die wegen eines fortgeschrittenen Karzinoms eine Resektion mit anschließender großvolumiger Radiochemotherapie (RCT) erhielten [4]. Weil gerade die Schluckfunktion, wie hier gezeigt, die Lebensqualität erheblich beeinflusst, ist ihr Erhalt essenziell. Tang et al. konnten zeigen, dass dies mit der Aussparung eindeutig definierbarer Lymphknotenstationen bei Patienten mit Nasopharynxkarzinomen gelingt, ohne dass es zu Einbußen der Tumorkontrolle kommt. Zusätzlich offenbaren ihre Untersuchungen, dass selbst ein Verzicht auf zu bestrahlendes Volumen unterhalb des Cartilago cricoidea die Schluckfunktion günstig gestaltet, selbst wenn proximal davon Dosen appliziert werden, die ungünstig für die Schluckfunktion sind [5].

Der randomisierten Interventionsstudie vorausgegangen war eine akribische Analyse der Verteilungsmuster der Lymphknotenmetastasierung an fast tausend Patienten mithilfe der MRT [2]. Sie bildete die Basis für eine Serie von 54 Patienten mit partieller Halsbestrahlung, die eine 98 %ige regionale Tumorkontrolle zeigte [6], und führte in letzter Konsequenz zur Durchführung einer randomisierten Phase-III-Studie, die nun auch eindeutig zeigt, dass dieses reduzierte Bestrahlungsvolumen trotzdem zu einer gleichwertigen und hohen Tumorkontrolle führt. Noch heute finden sich erstaunlich wenige von diesen essenziellen Analysen zur Verteilung von Lymphknotenmetastasen und Rezidiven bei jedweder Tumorentität, die unsere Festlegung der Bestrahlungsvolumina so nachhaltig beeinflussen. Hier ist eine wahre Fundgrube, die natürliche und künstliche Intelligenz beflügeln kann.

Auf den ersten Blick schwer erklärlich scheint das marginal bessere Gesamtüberleben und krankheitsspezifische Überleben in der Gruppe mit der dosisreduzierten Bestrahlung. Bei genauerer Betrachtung der Daten könnte dies durch die Tatsache bedingt sein, dass sich in der Gruppe der Patienten mit bilateraler Bestrahlung mehr Patienten mit T3/T4-Tumoren und ältere Patienten befanden als in der Gruppe der volumenreduzierten RT. Allerdings ändert dieser Aspekt nichts an der Tatsache, dass die volumenreduzierte Bestrahlung in dem eingeschlossenen Patientenkollektiv, welches in Europa sicher eine Rarität darstellen dürfte, bedenkenlos empfohlen werden kann. Wofür aus unserer Sicht derzeit keine hinreichenden Beweise vorliegen, ist der Verzicht auf die Bestrahlung der kontralateralen proximalen Lymphknotenstationen, auch nicht bei einseitigem Nasopharynxkarzinom. Aber das bleibt noch nachträglich zu untersuchen.

### Fazit

Die Daten der hier kommentierten Studie sind so eindeutig, dass in vielen Fällen auf eine Bestrahlung von Level IV und Vb verzichtet werden kann, und zwar dann, wenn ein Schmincke-Tumor (WHO-Typ III) vorliegt, ein begrenzter Lymphknotenbefall cN1 nach TNM-Klassifikation/Version 7, und wenn eine MRT des Halses zur Bestrahlungsplanung vorgenommen wurde. Die Aussagen der Studie gelten nicht für WHO-Grad-I- und -II-Tumoren und nicht für den bilateralen Lymphknotenbefall. Das heißt aus unserer Sicht auch nicht für Patienten nach Lymphknotenexstirpation, aber schon, wenn im Therapieplan eine hinreichend dosierte Chemotherapie vorgesehen und auch durchgeführt wird. Gerade Schmincke-Tumoren gelten ja neben ihrer Sensibilität für eine Strahlentherapie auch als äußerst chemosensibel. Bekanntermaßen reduziert die Chemotherapie die Rate der Mikrometastasen bei Nasopharynxkarzinomen um 15 % ab dem N1-Stadium [7]. Ein Effekt, der die Sicherheit der volumenreduzierten Bestrahlung sicher positiv beeinflusst.

*Marlen Haderlein und Sabine Semrau, Erlangen*
